# Roles of macrophages in tumor development: a spatiotemporal perspective

**DOI:** 10.1038/s41423-023-01061-6

**Published:** 2023-07-10

**Authors:** Mathilde Bied, William W. Ho, Florent Ginhoux, Camille Blériot

**Affiliations:** 1 Institut Gustave Roussy, INSERM U1015, Villejuif, France; 2 Singapore Immunology Network (SIgN), Agency for Science, Technology and Research (A∗STAR), Singapore, Singapore; 3 Shanghai Institute of Immunology, Shanghai JiaoTong University School of Medicine, Shanghai, China; 4 Translational Immunology Institute, SingHealth Duke-NUS, Singapore, Singapore; 5 Institut Necker des Enfants Malades, INSERM, CNRS, Université Paris Cité, Paris, France

**Keywords:** Macrophages, Tumor associated macrophages, Tumor microenvironment, Metastasis, Phagocytes, Immunosurveillance

## Abstract

Macrophages are critical regulators of tissue homeostasis but are also abundant in the tumor microenvironment (TME). In both primary tumors and metastases, such tumor-associated macrophages (TAMs) seem to support tumor development. While we know that TAMs are the dominant immune cells in the TME, their vast heterogeneity and associated functions are only just being unraveled. In this review, we outline the various known TAM populations found thus far and delineate their specialized roles associated with the main stages of cancer progression. We discuss how macrophages may prime the premetastatic niche to enable the growth of a metastasis and then how subsequent metastasis-associated macrophages can support secondary tumor growth. Finally, we speculate on the challenges that remain to be overcome in TAM research.

## Introduction

Macrophages are tissue-resident immune cells that emerge from multiple waves of hematopoiesis during embryonic development to seed their organs of residency [1]. In mice, primitive myeloid progenitors arise in the yolk sac around embryonic Day E7-8 and give rise to microglia, which are self-maintained locally from this initial seeding, independent of circulating monocytes [2]. A second semi-definitive wave of hematopoiesis starts at E8.25 and gives rise to progenitors that transiently shelter in the fetal liver, differentiate into monocyte-like cells [3, 4] that subsequently colonize fetal tissues to give rise to these cells are known as resident tissue macrophages (RTMs). A final, third wave of hematopoietic precursors emerges from the aorta-gonado-mesonephros region at E10.5 and leads to the generation of hematopoietic stem cells that will later establish definitive hematopoiesis in the fetal liver and then in the bone marrow. Monocytes from this third wave are recruited to tissues from late embryonic stages to adulthood, thus somewhat diluting the preexisting embryonic RTMs in a tissue-dependent manner [5]. Thus, contrary to early consensus [6], many RTMs found in adult tissues are long-lived cells with embryonic origins.

During organogenesis, macrophages undergo tissue imprinting whereby embryonic progenitors first acquire a core macrophage differentiation program including pattern recognition and cytokine receptors [7]. Then, tissue-specific programs emerge during embryonic development with the differential activation of transcription factors and gene networks [7]. Such tissue imprinting is not an event restricted to embryonic development: as we draw on in this review, the relatively long lifespan of RTMs means that they are inevitably exposed to both non-homeostatic events, such as inflammation or infection, and systemic signals. The somewhat continuous imprinting that ensues as a result of a dynamically altered niche can lead to RTM dysregulation, which in turn might favor oncogenesis [8].

**Fig. 1:**
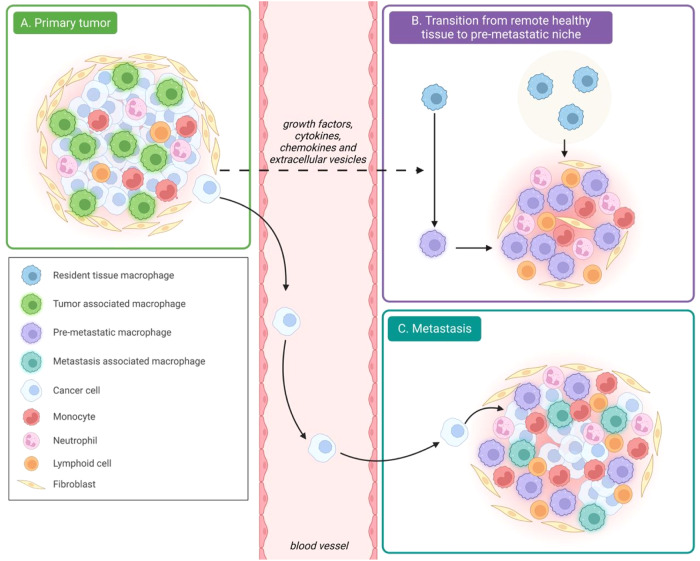
Macrophages involved in tumor growth and metastasis. **A** In the primary tumor, tumor-associated macrophages favor tumor development. **B** Tissue-resident macrophages from distant tissues shape the pre-metastatic niche upon stimulation with tumor-derived factors such as extracellular vesicles to prepare cancer cell colonization. **C** As the cancer cells colonize the pre-metastatic niche, recruited monocytes give rise to metastatic-associated macrophages that fuel metastatic progression

In the most basic sense, oncogenesis occurs as a result of an accumulation of mutations in oncogenes that permit normal cells to overcome restrictions on cellular replication such that they can grow without restraint to form a tumor. However, it is not only the tumor that has pathological consequences; other components that form the tumor microenvironment (TME) influence oncogenesis and cancer progression [9]. The TME comprises the blood and lymphatic vessels, extracellular matrix (ECM), and distinct host cells, including fibroblasts and immune cells, in the immediate ecosystem that surrounds the tumor, in addition to the tumor cells themselves [10]. Among the immune cellular components of the TME, macrophages have received particular attention. These so-called tumor-associated macrophages (TAMs) are typically the most abundant immune population within the TME, and their abundance is in fact now leveraged as a diagnostic marker, as it often correlates with prognosis [11–13]. In this review, we discuss the recent advances made in clarifying the roles of various populations of TAMs at key stages of cancer progression from tumor initiation to metastasis (Fig. 1).

## Macrophage diversity in the cancer context

Macrophage diversity is broadly conceptualized through the prism of two antagonistic polarization states: pro-inflammatory M1 and anti-inflammatory M2 macrophages [14]. Upon the identification of TAMs in the TME, a similar paradigm was adopted with immunosuppressive TAMs clinically associated with a poor prognosis, considered M2 macrophages [15–17]. A therapeutic extension of this view has culminated in attempts to repolarize TAMs from the M2 to M1 state by acting on key modulators of macrophage biology, such as colony-stimulating factor 1 [18, 19] or IFN-γ [20].

### Tumor-associated RTMs and monocyte-derived TAMs

Although TAMs undoubtedly exhibit immunosuppressive properties, the M1/M2 paradigm does not fully reflect the extent of macrophage and TAM heterogeneity [21] and the different states of macrophage activation uncovered as a result of recent studies using single-cell and lineage-tracing technologies [22–27] (Table 1). Concerning their ontogeny, most TAMs are derived from circulating monocytes [28] and can almost completely overcome the pre-existing population of embryonically derived RTMs. A small population of native embryonic macrophages can, however, persist in the TME and have distinct functions from TAMs, including specific remodeling of the extracellular matrix [29]. More strikingly, a differential location within the TME has been revealed for pre-existing tumor-associated RTMs and monocyte-derived TAMs in various cancers, including breast cancer [30], non-small cell lung carcinoma [31], and glioma [32]. In both lung cancer [31] and glioma [32], embryonic-derived RTMs are found preferentially at the periphery of the tumor, while monocyte-derived TAMs infiltrate the tumor core. Nevertheless, monocyte-derived HO-1^+^ TAMs have also been shown to preferentially localize at the invasive margins of primary tumors and metastases in the MN-MCA1 murine model of cancer [33], therefore arguing for disease-specific localization of ontogenically distinct TAM populations. Altogether, these results suggest that such distinction between tumor-associated RTMs and monocyte-derived TAMs should be made when considering TAM identities and functions.

**Table 1:** Major TAM populations and their specificities

TAM populations as defined in [22]	Species	Surface markers	Cancer	Functions	References
**IL4I1**^**+**^	Human Mouse	PD-L1, PD-L2, IDO1 To be defined	Colorectal Hepatocellular Glioblastoma Melanoma	Antigen presentation Phagosome maturation Treg recruitment T-cell suppression	[22, 134–137]
**TREM2**^**+**^	Human Mouse	TREM2, APOE, CD63, CD9 CADM1, CX3CR1, CD63, CD36	Colorectal Breast Glioblastoma Melanoma Squamous cell carcinoma Skin carcinoma	Lipid metabolism Matrix remodeling Immunosuppression Cancer cell proliferation	[22, 30, 136–140]
**FOLR2**^**+**^	Human Mouse	FOLR2, CD163, LYVE1, CD206 FOLR2, CD206, TIM4, LYVE1	Breast Hepatocellular	CD8^+^ T-cell infiltration	[22, 30, 141]
**FTL**^**+**^	Human Mouse	CD52, CXCR4, CD163 TIE2, CXCR4,	Colorectal Glioblastoma	Angiogenesis	[22, 134, 136, 137, 139]
**Proliferating**	Human Mouse	MKI67, TOP2A MKI67, TOP2A	Colorectal Glioblastoma	Cell cycle Proliferation	[22, 134, 136, 137, 139]

*Adapted from* [133]

## TAM function in primary tumors

**Fig. 2:**
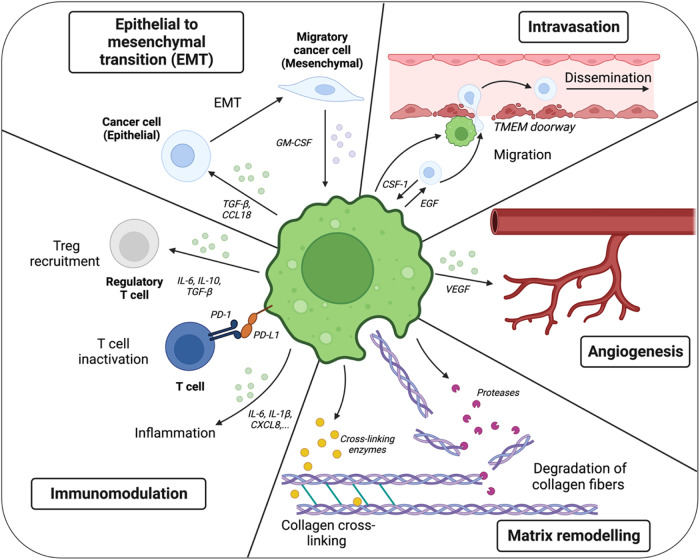
Tumor-associated macrophages favor tumor development through different functions. TAMs have various roles in tumorigenesis and as such, interact closely with cancer cells and the TME. TAMs create a pro-tumoral immune environment by: inactivating cytotoxic T cells through PD-L1 expression; and producing various cytokines to recruit regulatory T cells (IL-6, IL-10 and TGF-ß) and create an inflammatory milieu (IL-6, IL- 1ß, CXCL8). TAMs shape the extracellular matrix by producing proteases such as matrix metalloproteinases or cathepsins that degrade collagen fibers and ensure their turnover. TAMs also produce cross-linking enzymes that modulate the stiffness of the extracellular matrix. TAM-secreted VEGF promotes angiogenesis that facilitates tumor progression as well as metastasis. TAMs migrate with cancer cells to blood vessel where they create openings known as “TMEM doorways”, allowing cancer cells to disseminate in the circulation. Finally, TAMs produce TGF-ß and CCL18 that have a role in epithelia to mesenchymal transition, allowing cancer cells to migrate. Mesenchymal cells promote TAM activation through GM-CSF production

TAMs were originally considered remnants of an abortive immune response against the tumor [34]. However, in 2001, the Jeffrey Pollard group showed that mice with a recessive null mutation in the colony-stimulating factor 1 gene (*Csf1*^*op*^), the major macrophage growth factor, and genetically modified to develop mammary cancer had a delay in the development of metastatic carcinomas, therefore showing the involvement of macrophages in malignant progression of breast cancer [35]. These seminal findings have led to investigations into the mechanisms of these pro-tumoral roles of macrophages. We now know that within the TME, TAMs have several supporting functions that promote tumor development (Fig. 2), which we describe below.

### Vascularization

TAMs promote vascularization to supply oxygen and nutrients to cancer cells in the well-described process of angiogenesis [36]. Numerous investigations into this process have led to the detection of angiogenesis-promoting molecules produced by TAMs, including vascular endothelial growth factor-A (VEGF-A) in the context of non-small cell lung cancer [37] and adrenomedullin in melanoma [38]. Of note, this angiogenesis-promoting property is also observed in macrophages across healthy embryonic development [39]; therefore, we might speculate that this feature represents a function acquired by macrophages early in evolution, which reappears in these two different yet comparable contexts [40]. As a consequence, VEGF/VEGF receptor-targeting compounds are emerging as very promising therapies and are starting to be used notably to treat non-small cell lung carcinomas [41].

### Inflammation

Another prominent function of TAMs in primary tumors is their role in establishing and maintaining an inflammatory environment. Examples of such TAM-derived inflammatory factors favoring tumor development are multiple and include CXCL8 in endometrial cancer [42], IL-6 in breast cancer [43], and IL-1ß in pancreatic cancer [44]. While this proinflammatory profile is supposed to support an active immune response against tumors, the remarkable plasticity of TAMs makes them more often associated with immunosuppression. As such, TAMs have the capacity to promote regulatory T-cell (T_reg_) recruitment. This phenomenon has been highlighted in ovarian cancer [45], nasopharyngeal carcinoma [46], and liver cancer [47], where these T_regs_ can then deactivate cytotoxic T cells directed against tumor cells [48]. TAMs can also directly promote cytotoxic T-cell exhaustion [30, 49, 50], and many current immunotherapies aim to reactivate antitumoral cytotoxic T cells by inhibiting the PD-1/PD-L1 immune checkpoint pathway [51]. It is worth noting, however, that TAMs also express PD-1 [52] or PD-L1 [22] and could therefore be considered off-targets/second targets of current protocols using pembrolizumab or nivolumab. The impact of such indirect TAM targeting on patient responses to treatment is largely unknown but should be taken into consideration in future studies, particularly as the effectiveness of current immunotherapies is variable. Nevertheless, the apparent heterogeneity in patient responses to treatment is likely mediated, in part, by TAMs. For example, macrophage recruitment is enhanced in patients with prostate cancer treated with androgen blockade therapy, and this recruitment subsequently contributes to tumor development. Those administered with anti-CSF-1 antibody in parallel, however, show an improved response to treatment [53]. In a similar manner, macrophage depletion with an anti-CSF-1 antibody reduces tumor growth in a mouse model with mammary gland tumors treated with radiotherapy [54].

### Epithelial to mesenchymal transition

TAMs also promote epithelial to mesenchymal transition (EMT), a process during which epithelial-like, early proliferating cancer cells lose the capacity for cell–cell adhesion and adopt a fibroblast-like phenotype with invasive and migratory properties [55, 56]. EMT ultimately later permits metastatic cell dissemination. At the molecular level, EMT is orchestrated by the transcription factors zinc-finger E-box binding homeobox factor 1 (ZEB1) [57, 58], Snail [59, 60] and Twist [61] (reviewed in [62]). TAMs can regulate these EMT-modulating factors through their secretome [55, 63]. For example, TAM-produced tumor necrosis factor (TNF)-α stabilizes Snail through NF-kB signaling [64], while TAM-produced TGF-β induces Snail and ZEB1 expression by activating the β-catenin pathways [65–67]. Moreover, mesenchymal cell production of GM-CSF induces TAM activation and CCL18 production and further promotes EMT in a positive feedback loop [68].

### ECM remodeling

TAMs are also involved in active ECM remodeling, collaborating notably with cancer-associated fibroblasts (CAFs) to promote tumor cell intravasation [69]. Indeed, tumors often display a dense ECM that notably impairs drug penetration, limiting treatment efficacy and resulting in more metastases [70, 71]. TAMs express and secrete various membrane-associated proteases that degrade ECM collagen fibers, such as matrix metalloproteinases (MMPs) [72, 73], secreted protein acidic and rich in cysteine [74], and cathepsins [69, 73]. Once degraded, TAMs mediate collagen fragment turnover via phagocytosis and degradation in the lysosome by cathepsins [69]. TAMs, by producing cross-linking enzymes from the lysyl hydroxylase (LH) family, such as LH2 in triple-negative breast cancer [75], also increase ECM stiffness, which promotes tumor progression and metastasis by mechanical forces [76]. In addition, in models of lung adenocarcinoma and breast cancer, a subset of TAMs expressing fibroblast activating protein (FAP)-α, which acts both as a signaling protein for CAFs and as a collagenase, and heme oxygenase (HO)-1 was found to be associated with ECM remodeling [77, 78]. Altogether, these observations suggest that similar mechanisms are involved in both wound healing and tumor formation, in line with the famous statement that tumors are “wounds that do not heal” [79].

### Intravasation

EMT and ECM remodeling precede the intravasation of tumor cells into the circulation and their subsequent dissemination to distal organs. This key event in metastasis formation occurs at sites known as “tumor microenvironment metastasis (TMEM) doorways”, characterized by the dynamic association between one endothelial cell, one TAM and one cancer cell [80–82]. TAMs from the TMEM doorway arise from recruited monocytes that become CXCR4^+^ TAMs upon TGF-β stimulation in the TME. Attracted by fibroblast-derived CXCL12, these TAMs migrate toward the vascular niche, where they adopt a perivascular TAM phenotype and disrupt the junctions between endothelial cells, which allow tumor cells to intravasate into the circulation [83–85]. Of note, TMEM density in tumors has been linked with increased metastatic burden and could be used as a tool for predicting the occurrence of metastasis [80, 86].

In addition, activation of a paracrine loop also allows cancer cells that produce CSF-1 and TAMs that produce EGF to migrate together toward TMEM doorways. Thus, blocking CSF-1 or EGF receptors reduces cancer cell migration and invasiveness in breast cancer rodent models [87]. Furthermore, IL-4-producing T_H__2_-CD4^+^ T cells stimulate EGF production by TAMs, and depletion of CD4 + T cells or IL-4-neutralizing antibody treatment reduces the metastatic burden [88]. Collectively, these examples demonstrate the crucial role of macrophages in initiating the metastatic process by favoring the migration and intravasation of cancer cells into the blood circulation.

## Roles of RTMs in shaping the pre-metastatic niche

**Fig. 3:**
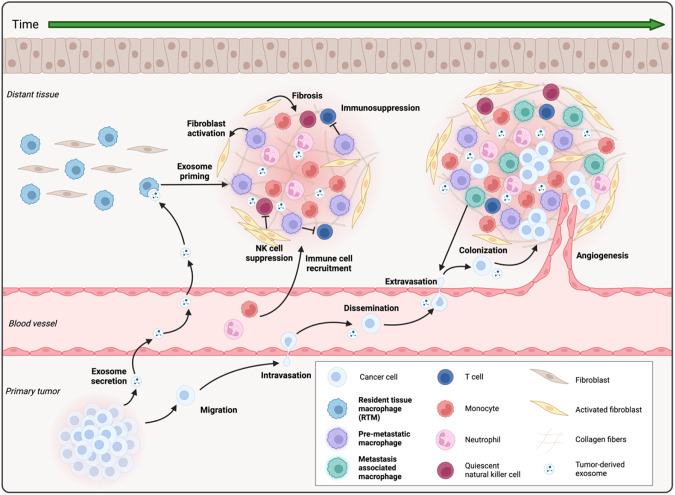
Pre-metastatic niche formation. Exosomes from the primary tumor reach distant tissues by trafficking through blood vessels. Once they arrive at the tissue site, they are engulfed by tissue resident macrophages (RTMs) cells, which triggers pre-metastatic niche formation. Activated RTMs are then able to activate fibroblasts, which in turn promote immune-cell recruitment and natural killer cell suppression. Activated RTMs also help establish a pro-tumoral environment by promoting inflammation and suppressing adaptive immune responses, thus creating a favorable environment for disseminating cancer cells. Activated RTMs maintain this favorable environment for metastatic growth when cancer cells start to colonize the pre-metastatic niche. Cancer cells favor monocyte recruitment that become metastasis-associated macrophages (MAMs). MAMs in turn favor metastasis progression through their role in cancer cell extravasation and T-cell suppression

In the late 19th century, Paget proposed the “seed and soil” theory of metastasis [89] in which tumor cells (the “seeds”) can only grow in a hospitable environment (the “soil”). While the nature of the “hospitable” environment remains to be defined, this theory suggests that changes occur in distant tissues before the arrival of cancer cells to ensure that the environment favors metastatic growth. These changes constitute the development of a “pre-metastatic niche” (Fig. 3). As key mediators of inflammation, macrophages produce various cytokines that directly prime naive tissue to welcome disseminated tumor cells [90].

### Influence of macrophage origin

Investigations are ongoing to understand how macrophages shape the premetastatic niche and whether their origins have a differential impact. This latter question has been approached using the *Cx3cr1*^CreERT2^-based fate mapping mouse model challenged with ovarian cancer cells, which has the capacity to form metastases in the omenta [91]. In this context, a specific subset of embryonic TIM4^+^ CD163^+^ omentum macrophages were shown to favor metastatic dissemination from ovaries to omenta, and their depletion resulted in reduced ascitic volume and metastatic invasion of this organ [91]. To date, the molecular mechanisms by which embryonic macrophages preferentially shape the pre-metastatic niche remain to be clarified, but their documented very long half-life could be a key parameter.

### Macrophage imprinting and extracellular vesicles

Mechanistically, pre-metastatic niche development and macrophage imprinting within that niche have been shown to be dependent on extracellular vesicles (EVs) that originate from the primary tumor and circulate within the blood. Of note, EVs have been classified based on their specific size and biogenesis and encompass microvesicles (150–1000 nm) arising from membrane budding, which are involved in local communication [92], and smaller exosomes (30–150 nm), which are derived from late endosomes and circulate over longer distances between tissues [92, 93]. EVs transport various cargos, such as RNAs, lipids, metabolites, or proteins, that they can transfer to other cell types to modulate their phenotype and functions. Through their distinct cargos, EVs impact the pre-metastatic niche through immune cell modulation, ECM remodeling and angiogenesis [94]. Findings derived from a seminal study from the group of David Lyden showed that integrins on the surface of tumor exosomes drive metastatic organotropism, as their patterns correlated with metastatic sites [93]. In the liver, for example, ITGαvβ5^+^ exosomes bind specifically to liver-resident macrophages (known as Kupffer cells), whereas ITGα6β4^+^ and ITGα6β1^+^ exosomes recognize lung fibroblasts and epithelial cells.

The mechanisms of action of tumor-derived EVs are only beginning to be elucidated. For example, macrophage migratory inhibiting factor (MIF) produced by primary pancreatic tumors and delivered by EVs remotely induces liver Kupffer cell production of TGF-β [95]. This process subsequently activates hepatic stellate cells (HSCs), which initiate liver fibrosis through fibronectin production, inducing the recruitment of inflammatory cells such as neutrophils and monocytes. Furthermore, activated HSCs also express CXCL12, which induces the quiescence of natural killer (NK) cells [96] and excludes CD8^+^ cytotoxic T cells from the pre-metastatic niche [97, 98]. The overall effect of this pathway is to reduce immunosurveillance of the pre-metastatic niche. Interestingly, monocyte-derived macrophages but not embryonically derived macrophages in the hepatic pre-metastatic niche secrete granulin, which serves to maintain HSC activation and liver fibrosis [99]. Further studies are needed to understand this observation, but as addressed earlier, it seems that macrophage origin affects the roles these cells play in determining metastasis.

In the liver, EV lipid cargo is handled by a specific subset of CD206^+^ Kupffer cells [100, 101], leading to upregulation of their expression of the fatty acid transporter CD36 and polarization toward an anti-inflammatory phenotype [102]. This phenotype favors immunosuppressive CD8^+^ T cells and improves the growth potential of disseminated tumor cells. Others have shown that EVs from lung adenocarcinoma notably induce upregulation of CD206, PD-L1 and GLUT1 by lymph node CD68^+^ macrophages. GLUT1 expression by macrophages increases their glucose uptake, and this glycolytic shift favors the establishment of the pre-metastatic niche [103]. In line with this, myeloid cells, including TAMs, have been shown to have the greatest capacity to take up glucose in the TME, a notably greater capacity than cancer cells [104], redefining the well-described Warburg effect. Coupled with the notion of the heterogeneity of TAM metabolic features [105], these findings argue that premetastatic niche priming relies on the metabolic capabilities of macrophages and promise groundbreaking discoveries with the increase in immunometabolism-related research.

These emerging findings, which place macrophages at the forefront of pre-metastatic niche establishment, can also be envisaged in the context of the macrophage network between distal tissues, which has been demonstrated to play a notable role in the context of myocardial infarction [106]. Indeed, it has been shown that macrophages from unrelated tissues such as lungs are activated after a heart-restricted challenge. The molecular mechanisms remain to be deciphered, but further studies could identify the actors involved in this phenomenon and assess their relevance in the context of cancer.

Finally, it should be noted that while EVs are scrutinized for their role in the priming of premetastatic niches, tumor cells can also prime distal macrophages via their release of free enzymes such as lysyl oxidase (LOX) [107].

## Roles of macrophages in maintaining metastases

Macrophages continue to support metastasis development after tumor cell migration has occurred. This is evident based on the finding that inhibiting TAM recruitment to a metastatic site results in a lower metastatic burden, as shown for lung [108–110] and liver [111] metastasis murine models. Specifically, in the liver, macrophages produce hepatic growth factor that binds to c-Met at the surface of migrating tumor cells [112], stopping their circulation and promoting their extravasation within the liver. In the lungs, a similar phenomenon occurs but is mediated by interactions between VCAM-1 at the surface of migrating tumor cells and integrin α4 at the surface of lung macrophages [109]. In addition, this interaction triggers the Ezrin-PI3K/Akt pathway in tumor cells, which confers some protection against proapoptotic cytokines [108].

### Metastasis-associated macrophages (MAMs)

Once the secondary tumor is established, macrophages deemed metastasis-associated macrophages (MAMs) in the literature [113] maintain immunosuppression by impairing cytotoxic T-cell activation. Specifically, and as mentioned earlier, EV-mediated priming of lung macrophages leads to a metabolic switch in these cells toward glycolytic respiration that produces lactate as a byproduct [103]. Lactate subsequently upregulates PD-L1 expression, blocking T-cell activation due to PD-1 engagement. Meanwhile, in the liver, macrophages induce systemic loss of T cells by triggering their apoptosis through the FAS-L pathway [114].

Many studies have described the recruitment of CCR2-expressing monocytes to the metastatic niche upon CCL2 production by stromal cells, which gives rise to MAMs [115, 116]. These monocytes might have different roles compared to RTMs present from the inception of the pre-metastatic niche. In MMTV-PyMT breast tumor-bearing mice, for example, monocyte-derived MAMs have a crucial role in cancer cell extravasation in the lung by producing VEGF-A [116], which can bind to the VEGF receptor on endothelial cells, thus inducing the remodeling of blood vessels at the metastatic site [117]. Monocyte-derived MAMs also seem to impact tumor-infiltrating lymphocytes in liver metastases of colorectal carcinoma. Specifically, a study in which colorectal cancer cells (MC38) were injected into the spleen of wild-type or CCR2 knockout mice showed that the knockout mice had a higher abundance of CD8^+^ and CD4^+^ lymphocytes and a reduced metastatic burden [115].

### Kupffer cells promote liver metastases

The liver is the most common site for metastasis, likely due to its dense blood vessel architecture, with the portal vein supplying a large amount of blood and hepatic sinusoids offering a secondary network with lower pressure and thus more time for migrating tumor cells to attach to the organ [118]. As shown in rats, Kupffer cells within the sinusoids limit these events through phagocytosis, clearing 90% of circulating tumor cells [119]. When Kupffer cells are overloaded, however, tumor cells can extravasate into the liver [120]. As mentioned earlier, Kupffer cells can also favor metastasis by activating HSCs and creating a fibrotic and inflammatory pre-metastatic niche that sustains tumor cell invasion [95, 96]. Kupffer cells also act as key drivers of liver metastatic tropism through their specific engulfment of tumor-derived exosomes [93]. Accordingly, depletion of Kupffer cells before the induction of liver metastasis resulted in an increased metastatic burden, while depletion of KCs after metastatic establishment reduced metastatic growth [120]. Of note, other populations of macrophages also populate the liver, such as capsular or lipid-associated macrophages (LAMs) [121–123]. These cells have only recently been described, and their role in cancer has not yet been fully characterized, although LAM accumulation in metastases has been reported [124].

### Lung macrophages modulate lung metastases

After the liver, the lungs constitute the second most frequent site of metastases. Exosomal priming of lung macrophages promotes the development of the pre-metastatic niche by inducing T-cell suppression [103] and neutrophil recruitment [125]. Lung macrophages also promote metastatic invasion by serving as anchors for circulating tumor cells, allowing their extravasation [108–110]. Again, heterogeneous macrophage populations with different features inhabit the lungs [126] and could have various roles in the metastasis of different primary tumors to this organ. For example, interstitial macrophages evolve over time in the metastatic niche, first exhibiting an antitumoral phenotype and later a protumoral phenotype, likely due to signals received from the stroma [103]. Alveolar macrophages also play a role in metastasis development, and notably, a subpopulation of lipid-laden Trem2^+^ macrophages display metabolic, immunosuppressive and matrix remodeling features that accumulate in metastases [127].

## Limits and future perspectives in TAM research

In this review, we have highlighted various facets of tumor-associated macrophage biology that influence different steps of cancer development. The versatility of TAM functions is evident; thus, it is difficult to identify one unified target that might be of clinical benefit [128–130]. The very limited efficacy of global approaches such as those targeting the CSF-1/CSF-1R or CCL2/CCR2 pathways illustrates the challenges faced. Therefore, refinement of our strategies is needed and is on-going, as exemplified by the recent results suggesting efficacy of a combination of a TREM2-specific antibody with the widely used anti-PD-1 antibody in different cancer models [131].

To argue for this improved consideration of TAM heterogeneity, we have discussed the extent of TAM heterogeneity, with TAMs actually encompassing spatiotemporally unrelated macrophage populations within primary tumors, distal healthy tissues and metastasis sites. It remains to be fully deciphered how fundamental determinants of macrophage biology, such as their origin, their local environment and the time spent in the tissues, differentially influence tumor progression in these three different contexts [132].

To tackle these fundamental questions, our methodology needs to evolve. Many studies have relied on mouse models of cancer thus far, but we should acknowledge the inherent limitations of these systems. Orthotopic models, such as the widely used canonical B16 melanoma model, are convenient and easily combinable with knock-in or knock-out animals; unfortunately, this type of model is quite different from the natural disease course of cancer. Indeed, while the primary TME can be more-or-less recapitulated depending on the models, these systems completely bypass the key step of pre-metastatic niche priming owing to their fast-developing nature. This feature disconnects these models from patient contexts in which, as previously stated, metastasis remains the main cause of death. Genetic models closer to what is observed in patients do exist but are usually less convenient due to their lower penetrance and often asynchronous tumor emergence, limiting reproducibility and the establishment of robust conclusions. In contrast, patient biopsies represent invaluable samples and are extensively used; however, disease genesis is difficult to determine from one end-point sample from one location, either the primary tumor or metastasis, and only limited information can be extracted from the analysis.

Considering these issues, meaningful alternatives are needed to better understand the roles of macrophages in every step of the disease process. There are many avenues to be explored, and the recent increase in single-cell omics technologies offering snapshots of tissue activity at an unprecedented resolution will no doubt enable the precise identification of targets during disease development. These approaches now need to be coupled with models that consider disease dynamics, from the initial acquisition of oncogenic mutations to metastasis and multiorgan failure. The most recently developed spatial transcriptomic technologies allow for the identification of pathways that are activated in TAMs but also in all the other cells from the TME while conserving its architecture. These technologies can even be applied to fixed samples, allowing the retrospective analysis of hundreds of thousands of samples from cancer patients stored in hospitals worldwide. The increase in immunometabolism research should also reveal novel insights into macrophage activity within the TME, which could lead to the development of a new generation of metabolite-targeted therapies to reprogram TAMs into anti-tumor cells. It is up to us to make fruitful use of this wealth of information to generate knowledge that will inform the precise design of innovative TAM-related immunotherapies.
